# Simultaneous EEG-fMRI reveals theta network alterations during reward feedback processing in borderline personality disorder

**DOI:** 10.1038/s41598-021-96209-7

**Published:** 2021-08-30

**Authors:** Paul A. Schauer, Jonas Rauh, Sarah V. Biedermann, Moritz Haaf, Saskia Steinmann, Gregor Leicht, Christoph Mulert

**Affiliations:** 1 Psychiatry Neuroimaging Branch, Department of Psychiatry and Psychotherapy, Center of Psychosocial Medicine, University Medical Center Hamburg-Eppendorf, Hamburg, Germany; 2 Department of Psychiatry and Psychotherapy, Center of Psychosocial Medicine, University Medical Center Hamburg-Eppendorf, Hamburg, Germany; 3 Department of Psychiatry and Psychotherapy, UKGM, Justus-Liebig- University Giessen, Giessen, Germany

**Keywords:** Reward, Psychiatric disorders

## Abstract

Previous studies using imaging techniques such as electroencephalography (EEG) or functional magnetic resonance imaging (fMRI) have identified neurophysiological markers of impaired feedback processing in patients with Borderline Personality Disorder (BPD). These mainly include reduced oscillatory activity in the theta frequency range in the EEG and altered activations in frontal and striatal regions in fMRI studies. The aim of the present study is to integrate these results using a coupling of simultaneously recorded EEG and fMRI. Simultaneous EEG (64-channel) and fMRI (3-Tesla Siemens Prisma) was recorded whilst participants (19 BPD patients and 18 controls) performed a gambling task. Data was analysed for the two imaging techniques separately as well as in a single-trial coupling of both modalities. Evoked theta oscillatory power as a response to loss feedback was reduced in BPD patients. EEG-fMRI coupling revealed an interaction between feedback valence and group in prefrontal regions centering in the dorsolateral prefrontal cortex (dlPFC), with healthy controls showing stronger modulation by theta responses during loss when compared to gain feedback and the opposite effect in BPD patients. Our results show multiple alterations in the processing of feedback in BPD, which were partly linked to impulsivity. The dlPFC was identified as the seed of theta-associated activation differences.

## Introduction

Personality disorders are among the most common psychiatric disorders with prevalences in recent studies ranging between 12%^1^ and 20%^2^ of overall population. Within the spectrum of personality disorders the Borderline Personality Disorder (BPD) stands out with high mortality rates^3^ and societal costs^4^. Patients with BPD show considerable impairments in different areas of everyday functioning, such as affect regulation, interpersonal relationships, impulse control and self-perception^5^ and often display psychiatric comorbidities^6^. In order to improve therapeutic interventions for this severe psychiatric disorder it is important to better understand the mechanisms underlying the impairments of patients suffering from BPD.

The reward system is considered to be a core player in the pathophysiology of BPD^7^. Many studies have tried to identify the main brain regions and mechanisms of the so-called reward network. EEG studies for example have identified markers of feedback processing that seem to be altered in BPD^8,9^. One well-established marker in the domain of event-related potentials (ERPs) is the feedback-related error negativity (fERN)^10^. It is characterised by a negative deflection in the EEG peaking around 250–300 ms after loss feedback^11^ and seems to be reduced in BPD^9^.

In addition, anomalies in so-called event-related oscillations (EROs) have been reported. Oscillatory activity has often been regarded as an important driver for controlling and coordinating neuronal responses and information transmission^12–14^. Neuronal oscillations have also previously been interpreted as a kind of “communication” between the different parts of the reward network^15^. Different frequency specific responses have been noted to gain and loss feedback in the beta and theta frequency ranges respectively^16,17^. These have previously been interpreted as correlates of phasic and tonic activity of dopaminergic neurons^15,18^. An EEG-fMRI study in healthy participants has located the beta- and theta-specific activity in subcortical and fronto-parietal areas, respectively^15^. Even though the processing of positive feedback (i.e. gain feedback) seems to be mainly undisturbed in BPD, several studies observed reduced responses in the theta frequency range to negative (i.e. loss) feedback in BPD^8,9^. These impairments have been associated with impulsiveness and with certain brain areas such as the dorsomedial prefrontal cortex (dmPFC) and the anterior cingulate cortex (ACC) using EEG source localisation^8^.

An alternative approach to investigating the reward network is the use of functional magnetic resonance imaging (fMRI). To the best of our knowledge so far only three studies have used fMRI to investigate possible alterations in the feedback processing of BPD^19–21^. Vega et al.^21^ found alterations in the orbitofrontal cortex related to non-suicidal self-injury, whilst Herbort et al.^20^ focused mainly on differences in striatal regions and their association with impulsivity, but stressed the need to investigate cortical regions (i.e. the ACC and insula) in more detail. This makes sense considering that Enzi et al.^19^ found prominent differences between BPD patients and controls within the ACC in addition to striatal regions. This is in line with another study investigating reward processing in Cluster B personality disorders (i.e. Antisocial and Borderline Personality Disorder), which found prefrontal regions including the ACC and dorsolateral prefrontal cortex (dlPFC) to be altered in the processing of feedback in this patient group^22^.

Even though the results of the aforementioned unimodal EEG- and fMRI-studies are interesting in their own right, it is clear that only a combination of these two methods could yield deeper insight into the interplay between reward-associated areas identified by means of fMRI and the EROs as a measure of functional interactions within the reward network. Whereas the EEG has an excellent temporal resolution it has limitations in the spatial domain due to the lack of a clear solution to the inverse problem of cortical source localisation^15^. The opposite applies to the fMRI, which has an excellent spatial resolution but suffers from constraints in the temporal domain due to slice timing. An approach to overcoming these limitations is the simultaneous recording of EEG and fMRI. This approach has been used in healthy subjects in three separate studies^15,23,24^, but not in investigating the alterations in feedback processing observed in BPD patients, so far.

The aim of the present study is to identify networks associated with the altered oscillatory responses to feedback processing in BPD using EEG-informed fMRI. Based on results of previous studies we expect reduced activity in the theta frequency range as a response to loss feedback in BPD patients. Considering the results of studies investigating the theta-network in healthy populations we expect these alterations to be linked to reduced activations in fronto-parietal regions including the ACC. In addition, we hypothesize that trait impulsiveness is negatively correlated to activity in these fronto-parietal regions.

## Results

Patients with BPD showed significantly higher impulsiveness scores assessed via the BIS^25^ when compared to healthy controls (*t*(35) = − 4.54; *p* < 0.001).

The reward feedback paradigm used in this study consisted of several distinct phases (see Fig. 3). During the “choice” phase of the paradigm participants were instructed to choose between a “high-risk” and a “low-risk” option (i.e. 25 and 5 points at stake, respectively) via button press (no significant difference between groups regarding reaction times: controls: 675 ms (SD = 161); patients: 717 ms (SD = 118)). The mean percentage of the high magnitude choice (i.e. participant chooses 25) was 55.2% (SD = 10.19) in the control group and 56.6% (SD = 11.52) in the patient group (no significant difference). Both groups chose the high magnitude option significantly more often (*F*(1,35) = 10.26; *p* < 0.01). In the “feedback” phase of the paradigm the participants were informed whether they had won or lost the chosen amount of points.

**Figure 1:**
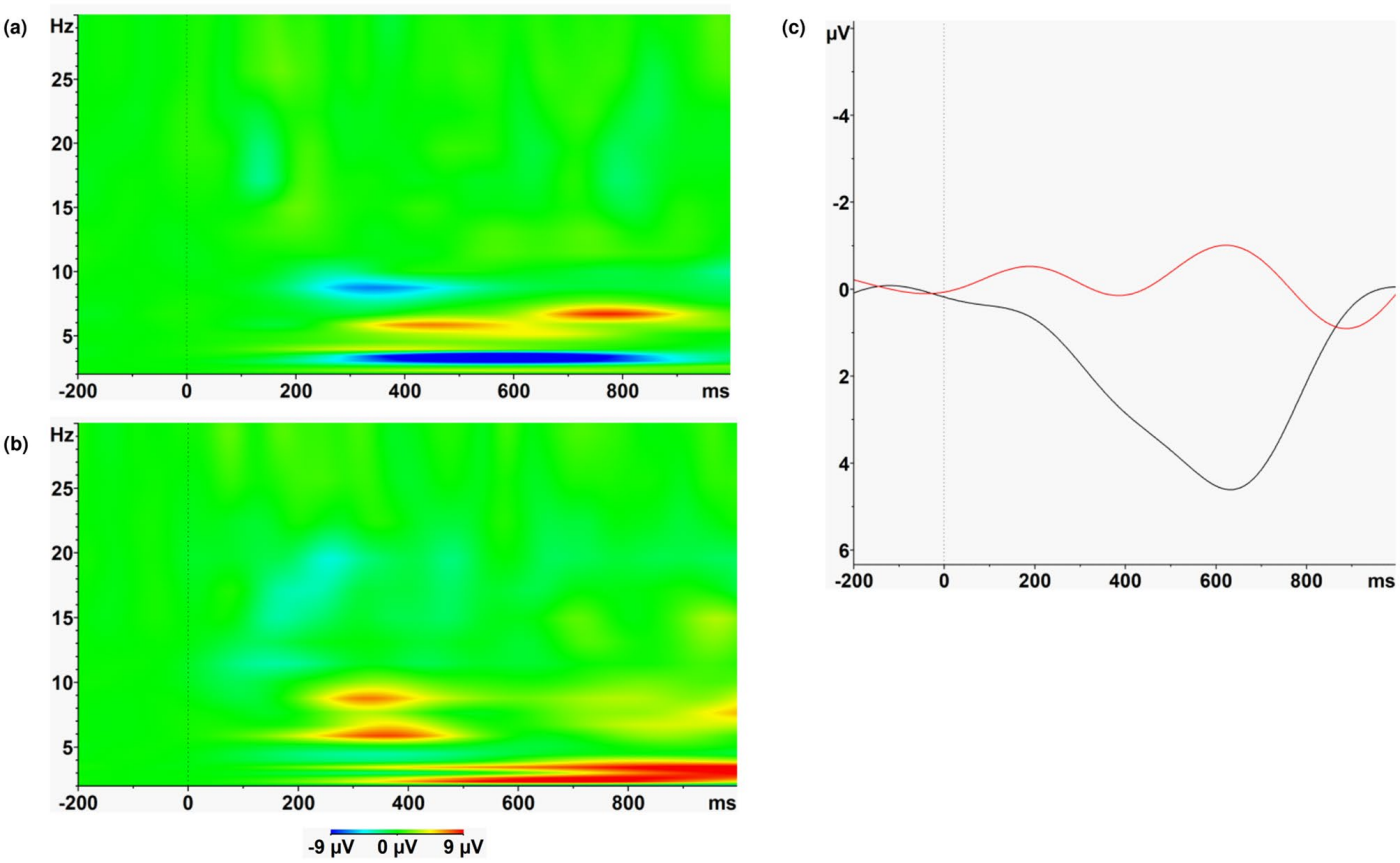
Time–frequency plots depicting the loss vs. gain differences in evoked oscillatory responses in the high magnitude condition in healthy controls (**a**) and BPD patients (**b**). Values are normed with respect to a 200 ms pre-stimulus baseline. (**c**) depicts the wavelet layer extraction for evoked theta power (central frequency: 5.1 Hz) for patients (red) and controls (black).

With respect to the EEG theta responses to these visual reward feedback stimuli the group x valence interaction reached significance (*F*(1,35) = 4.382; *p* = 0.044) with stronger increases in theta power after loss feedback in the control group compared to patients (see Fig. 1). No other effects reached significance (see Tables S1 and S2 in the supplementary material).

There were no significant differences regarding the latency of the theta peaks between groups, valence types or magnitude types (see Table S3 in the supplementary material).

With regard to the EEG high-beta responses to reward feedback no effects reached significance (see Tables S4 and S5 in the supplementary material).

**Figure 2:**
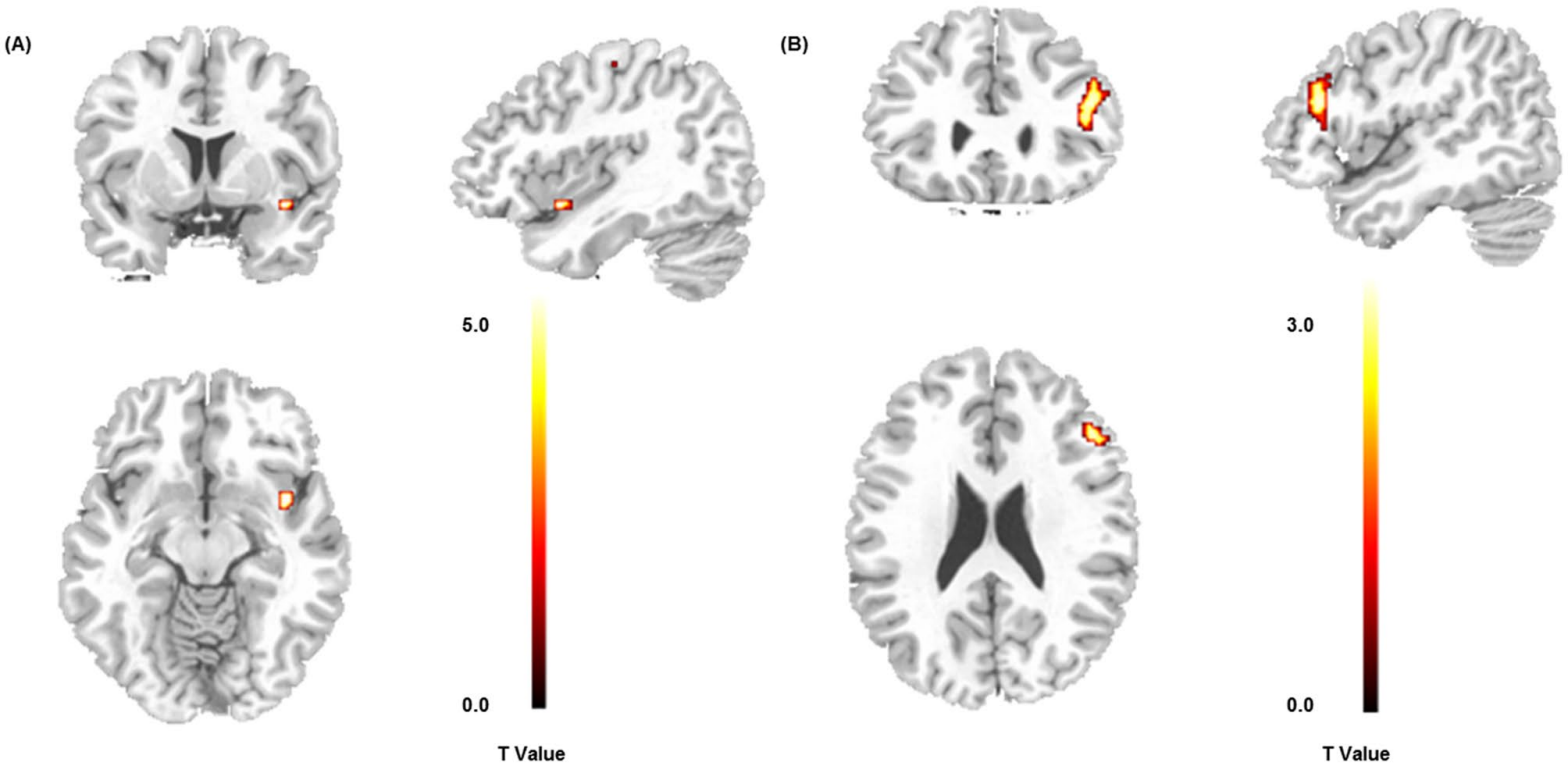
(**a**) Areas showing stronger BOLD-responses for controls vs. patients across all feedback conditions (regular fMRI; significance level: *p*(FWE) < 0.05). (**b**) Areas showing theta-band associated activation for the group × valence (loss) interaction (theta power coupling with BOLD activity; single-voxel *p* < 0.005, *k* ≥ 100). This figure was created using SPM12^45^ (Version 12; www.fil.ion.ucl.ac.uk/spm/software/spm12/) and MRIcro^49^ (Version 1.40; people.cas.sc.edu/rorden/mricro/).

For the group (controls > patients) contrast the fMRI analysis revealed significant activations in two clusters in the left anterior insula and the left postcentral gyrus following the reward feedback presentation (see Fig. 2a and Table 1). No other contrasts revealed significant activations in the fMRI whole-brain analysis.

**Table 1:** fMRI activations.

Anatomical Area	Coordinates	*p*(FWE)	Cluster Size	z-Score
*Controls* > *patients*				
L anterior Insula	-40 6 -12	0.005	21	5.15
L postcentral gyrus	-42 -20 56	0.021	5	4.73

Significance level: *p*(FWE) < 0.05.

fMRI, functional magnetic resonance imaging; FWE, family-wise error rate; L, left.

The fMRI region of interest (ROI) analysis revealed marginally significant main effects for the factors valence (*F*(1,35) = 4.09; *p* = 0.051) and group (*F*(1,35) = 3.71; *p* = 0.062) in the ACC ROI. Activations were stronger for loss compared to gain trials and controls compared to patients. No interactions between these factors were significant.

We used the single-trial theta response to the presentation of the feedback stimuli (frequency range: 4.4–5.8 Hz, maximum theta power within the time-frame of 200–900 ms following the stimulus) to inform a second fMRI analysis. This analysis revealed a significant group x valence (loss) interaction for a cluster centering in the left dlPFC (see Fig. 2b and Table 2). Controls showed a stronger modulation of the dlPFC activation by theta responses during loss compared to gain feedback; the opposite was true for BPD patients. No other contrasts revealed significant results in the EEG-informed fMRI whole-brain analysis.

**Table 2:** EEG-fMRI coupling (theta-associated activations).

Anatomical area	Coordinates	*p*(FWE)	Cluster size	z-Score
Group × valence(loss) interaction				
L middle frontal gyrus	− 46 28 20	0.929	108	3.09
L inferior frontal gyrus	− 44 28 12			2.90
L middle frontal gyrus	− 50 24 32			2.73

Significance level: *p* < 0.005 (uncorr.) and cluster extent *k* ≥ 100.

*EEG* electroencephalography, *fMRI* functional magnetic resonance imaging, *FWE* family-wise error rate, *L* left.

Moreover, there was no significant effect regarding the theta-specific activation of the ACC ROI.

In an explorative analysis looking for a relationship between our neurobiological and behavioral measures we found significant negative correlations between impulsiveness and general feedback activation (i.e. all four feedback conditions) in the anterior insula (*r* = − 0.327; *p* < 0.05) and the postcentral gyrus (*r* = − 0.326; *p* < 0.05) and the loss specific activation in the ACC (*r* = − 0.326; *p* < 0.05). The loss-specific activation in the theta-specific cluster in the dlPFC showed no significant correlation with impulsivity. None of the above correlations survived correction for multiple comparisons via FDR (false discovery rate). See Figures S3–S5 in the supplementary material.

## Discussion

The aim of the present study was to examine alterations in neurophysiological feedback processing of patients suffering from BPD in comparison to healthy controls. To this end we used single-trial coupling of simultaneously acquired EEG and fMRI data measured during performance of a gambling paradigm. Stand-alone fMRI analysis revealed alterations in feedback processing irrespective of feedback valence or magnitude between both groups in the anterior insula and postcentral gyrus, with both showing significantly stronger activation in healthy controls. Irrespective of group, loss feedback elicited stronger responses than gain feedback in the ACC. Regarding the EEG response we found a diminished response to loss feedback in the theta frequency range in BPD patients. Informing the fMRI analysis with single-trial power values of the theta oscillatory activity revealed an interaction between feedback valence and group in prefrontal regions including the dlPFC, with healthy controls showing stronger modulation by theta responses during loss when compared to gain feedback and the opposite effect in BPD patients. Moreover, general feedback activation in the anterior insula and postcentral gyrus and loss-specific activity in the ACC correlated negatively with impulsivity.

These results are in line with previous findings on a diminished response to loss feedback in the theta frequency range in BPD patients^8,9^. To the best of our knowledge this is the first time that the generators of this disturbed theta oscillatory response could be located in prefrontal areas using simultaneously acquired EEG and fMRI integrating the high temporal and spatial resolutions of these methods.

The reduced theta-modulated activation of the dlPFC underlying the reduced theta response to loss in BPD is the central finding of our study. This is in line with prior studies showing alterations in this region in cluster B personality disorders^22^ and identifying the dlPFC as part of a theta-specific network processing loss feedback in healthy participants^15^. This is especially interesting in the light of evidence on alterations in the theta frequency range related to prefrontal brain areas and loss feedback processing in other neuropsychiatric diseases such as schizophrenia^26^ and substance abuse disorder (SUD)^27^. Leicht et al.^26^ reported reduced theta band responses to loss feedback and related them to prefrontal dopamine deficits in schizophrenia. In accordance with this, a well-established model for reward processing in SUD proposed a regulating influence of prefrontal areas (including the dlPFC) on reward processing exerted through oscillations in the theta frequency range (Volkow and Morales)^27^. The authors of this article also proposed transcranial magnetic or electric stimulation of these areas as a potential therapeutic tool to strengthen executive control.

Thus, the results of our study might be able to contribute to the development of individualized non-invasive brain stimulation treatment strategies such as transcranial alternating current stimulation (tACS) of the dlPFC in BPD, as recently shown to be possible^28^ and successful^29^ in therapy-resistant obsessive–compulsive disorder. Such treatment approaches could strongly benefit from the possibility of detecting individual target brain regions as well as individual frequencies of interest in single subjects by means of simultaneous recordings of EEG and fMRI^30^.

A potential link between the observed alterations in the dlPFC, the anterior insula and the parietal cortex in BPD in our study might lie in a propensity for risk-taking. A meta-analysis of fMRI-studies showed risk-taking to be represented by activation in the anterior insula, dmPFC, dlPFC and parietal regions^31^. The authors of the study proposed a network of brain regions including the anterior insula as an emotional feedback region and the dmPFC acting as cognitive component, whilst dlPFC and parietal cortex supposedly integrate the information to form a decision. Additionally, the anterior insula was reported to be especially responsive to losses, which might indicate the potential role of this region in the feedback network.

Moreover, insula activation has often been linked to substance abuse disorders (SUD)^32^, which are known to be a common comorbidity in BPD^33^. Stimulation of the anterior insula in an animal model had an inhibitory effect on alcohol consumption^34^ suggesting the anterior insula as a potential target for brain stimulation in patients with SUD. The combined stimulation of insula and prefrontal cortex in humans resulted in reduced cigarette consumption^35^, whereas stimulation of the insula alone did not reduce alcohol drinking or craving in another study^36^. This is in line with a study showing a disturbed functional network for reward and cognitive control in SUD including prefrontal areas like the dlPFC^37^.

Whilst patients with comorbid SUD were excluded from the present study, it is interesting that both BPD and SUD seem to share certain overlaps regarding the pathophysiology in reward processing with alterations within similar brain regions such as the insula and dlPFC. To disentangle potential overlaps between the two disorders a next step would be to compare alterations between a BPD group without SUD and a BPD group with comorbid SUD.

Even though only marginally significant, our results are in line with studies attributing central mechanisms in the processing of loss feedback to the ACC^8^. These effects were also often associated with impulsivity^15^ and we were able to confirm this assumption. Regarding potential alterations in the ACC in BPD we were not able to show significant differences between the two groups, even though the effect again only trended towards significance. Future research could focus more specifically on this region to conclusively assess its part in the alterations of feedback processing found in BPD. Additionally it could be possible that bigger sample sizes are needed to reliably detect subtle alterations in this region.

Contrary to our hypothesis, we found no significant effects of gain processing in the fMRI analysis. This is surprising in view of results of previous studies^19,20^ and might be due to the fact, that reward anticipation as investigated in these studies has been shown to activate partly different areas compared to reward outcome processing as investigated in our study. Namely, reward anticipation evokes stronger striatal responses, whereas reward outcome processing involves the frontal cortex^38^. However, there are contrary results^15^. In further studies, it might be interesting to disentangle different aspects of reward processing investigating underlying genetic aspects of reward processing, dopamine system and electrophysiological parameters^39^.

Limitations of our study include the relatively small sample size, which might have prevented the detection of more subtle group and valence differences in reward feedback processing. Moreover, our relatively simple gambling paradigm did not allow for a comparison of the responses to gain and loss to a neutral condition and we were not able to monitor participants’ expectations. Therefore we were not able to distinguish the response to loss from a response resulting from a prediction error. Furthermore, it is possible that artifacts from the light, ventilation system or the MRI scanners helium pump generated artifacts that might affect data quality, even though we used pilot data to check for possible artifacts especially from the helium pump and found no such effects (see Figure S6 in the supplementary material). Lastly, even though we included the six motion parameters in our fMRI general linear model (GLM) in order to control for possible motion artefacts, it is possible that certain abrupt movements by the participants might still have influenced data quality*.*

In summary, patients with BPD showed altered responses to loss feedback in the theta frequency range of the EEG. Using fMRI we found reduced activity within the anterior insula and parietal regions during reward feedback processing in BPD. Activations in the insula, postcentral gyrus and the ACC showed negative correlations with participants’ impulsiveness ratings. Finally, the coupling of theta power with the BOLD response revealed a reduced theta-modulated activity within a cluster centering in the dlPFC in BPD. Whilst controls elicited stronger theta-modulated BOLD responses to loss, the opposite was true for patients with BPD. These results could be used to determine possible targets for brain stimulation to alleviate the burden of patients suffering from BPD.

## Methods

### Participants

The total sample for this study consisted of 21 BPD patients and 22 healthy controls. Patients were recruited from the in- and outpatient clinics of the Department for Psychiatry and Psychotherapy, University Hospital Hamburg-Eppendorf, whereas healthy controls were recruited through advertisements and word-of-mouth. The study was conducted in accordance with the Declaration of Helsinki of 1975, as revised in 2008, and was approved by the local ethics committee of the Medical Council of Hamburg. All participants provided written informed consent prior to inclusion in the study.

Patients were required to fulfill criteria of BPD according to DSM-5. The Mini International Neuropsychiatric Interview^40^ and expert ratings from clinicians working in the Department for Psychiatry and Psychotherapy, University Hospital Hamburg-Eppendorf, were used to establish the diagnosis of BPD and assess Axis I comorbidities in patients. In order to minimize the effects of comorbid disorders associated with reward system dysfunction, patients were excluded from the study if they presented a current depressive episode or a score of 12 or higher on the Montgomery–Asberg Depression Rating Scale (MADRS)^41^, a lifetime diagnosis of alcohol or drug dependence, or alcohol or drug abuse in the past year, or a lifetime diagnosis of psychotic or bipolar disorder. Further exclusion criteria for all subjects were neurological and developmental disorders, intellectual disabilities, presence of uncorrected visual problems or hearing loss. In healthy controls, additional exclusion criteria were a family history of psychotic disorders or personal history of any psychiatric disorder or treatment.

Trait impulsivity in participants was assessed via the Barratt Impulsiveness Scale (BIS)^25^.

Participants were reimbursed for study participation with 8 € per hour of study participation.

Two patients had to be excluded from the analysis: one due to an intellectual disability diagnosed after study participation, one due to not properly conducting the paradigm. Moreover, four healthy controls had to be excluded: two due to the wish to stop the EEG-fMRI-measurement, two due to bad EEG data quality (i.e. substantial artifacts could not be removed by the methods described). Thus, 19 patients and 18 healthy controls were included in the final analysis (see Table 3).

**Table 3:** Characteristics of the two participant groups.

	Healthy controls N = 18 *(22)*		BPD N = 19 *(21)*		χ 2 / t	*P*
	N		N			
**Gender**					1.08	p = 0.30
Female	17 *(21)*		19 *(19)*			
Male	1 *(1)*		0 *(2)*			
**Education**					1.12	p = 0.57
Special School	0 *(0)*		0 *(1)*			
Secondary school	0 *(0)*		1 *(1)*			
Secondary modern school	4 *(5)*		5 *(5)*			
General higher education entrance qualification	14 *(17)*		13 *(14)*			
	Mean	SD	Mean	SD		
Age	26.78 *(26.00)*	5.53 *(5.35)*	27.47 *(27.29)*	6.52 *(6.31)*	− 0.35	p = 0.73
BIS-11 total score	56.28 *(56.14)*	8.27 *(7.54)*	70.37 *(70.24)*	10.40 *(9.88)*	− 4.54	**p < 0.001**
BSL-23 total score	5.35 *(5.35)*	4.09 *(3.79)*	50.95 *(50.10)*	17.67 *(17.72)*	− 10.38	**p < 0.001**

*BPD* borderline personality disorder, *BIS-11* Barratt Impulsiveness Scale, *BSL-23* Borderline Symptom List.

### Paradigm

**Figure 3:**
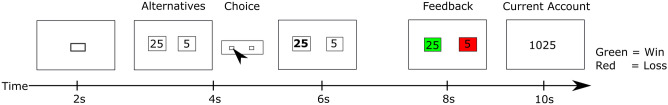
Depiction of the procedure of the gambling task adapted by us from Gehring and Willoughby^42^. This figure was created using Inkscape^50^ (Version 0.92; www.inkscape.org).

Participants performed a computerized two-choice gambling task adapted from Gehring and Willoughby^42^ and used in previous studies by our group and others^9,16,17^. Each trial began with the presentation of a fixation square for 2 s, followed by two numbers (5 and 25) on a computer screen (randomized left–right order) for 2 s. Within this time, participants were required to select one of the two numbers per button press. Two seconds after trial onset the chosen number was set to bold, if the participant had failed to answer via button press the trial was discarded. After a further delay of 2 s one of the two numbers randomly turned green and the other red indicating win or loss of the chosen amount respectively. Thus, the feedback stimulus varied along two dimensions, valence (positive vs. negative feedback) and magnitude (5 vs. 25 points). The feedback stimulus was displayed on the screen for 2 s followed by a display of the current account status for 2 s (see Fig. 3).

The Presentation software (Version 19, Neurobehavioral Systems, Albany (CA), United States) was used for stimulus presentation. Participants were instructed in a standardized way to choose freely between the two presented numbers (5 or 25) in every trial and to gain as many points as possible during each block. Participants were not required to choose 5 and 25 in an equal ratio. The occurrence of loss and gain events was maintained at equal probability (50% each). The paradigm consisted of four blocks (100 trials each, trial duration: 8 s) and was approx. 75 min of length. The inter-trial interval consisted of the presentation of a fixation square for 2 s. The inter-block interval lasted three minutes.

The participants viewed the paradigm via a mirror that was mounted on the head coil in such a way that they were able to see a screen on which the paradigm was presented. This in turn was placed directly behind the scanner.

### EEG recording

EEG was recorded during fMRI acquisition using the BrainVision Recorder software (Version 1.21, Brain Products, Gilching, Germany) and MR-compatible amplifiers (BrainAmp MRplus, Brain Products). An electrode cap (BrainCapMR, Brain Products) with 62 passive sintered silver/silver chloride EEG electrodes was used. Electrodes were positioned according to a modified 10/10 system. Additional electrodes were used for recordings of eye movements and the electrocardiogram. The electrodes FCz and AFz served as reference and ground, respectively. The amplifiers were placed within the scanner tube approx. 30 cm behind the head coil. To avoid artifacts from the reverberations by the MRI the cable connecting the electrode cap and the amplifiers and the amplifiers themselves were fixated with sandbags on foam cushions. Electrode skin impedance was kept below 10 kΩ. The data were collected with a sampling rate of 5000 Hz and an amplitude resolution of 0.5 μV. The helium pump, ventilation and lights were not switched off during data acquisition.

### EEG pre-processing

Offline preprocessing was performed using the Brain Vision Analyzer software (Version 2.1, Brain Products). In order to correct for the continuous MR-Artifact a sliding average template was created from 21 intervals using baseline correction. Data was then filtered with a 50 Hz low-pass (slope 12 dB/oct) and a 0.1 Hz high-pass (slope 48 dB/oct) butterworth zero phase filter and resampled to a 500 Hz sampling rate. Cardioballistic artifacts were corrected by subtraction of a semi-automatically detected template in the ECG-channel. An automatic raw data inspection was applied identifying voltage steps of more than 50 μV/ms, a difference higher than 250 μV between the highest and lowest values within a 200 ms timeframe and activity below 0.5 μV. Excluding segments of 200 ms around these artifacts an independent component analysis (infomax-gradient-extended biased algorithm) was conducted in order to eliminate further artifacts stemming from blinks, eye-movements, residual gradient artifacts, head movements and muscle activity based on their power-spectrum, topography and time-course. Data was then re-referenced to a common average reference. Subsequently, the data was segmented relative to the feedback stimulus starting 1800 ms before stimulus presentation and ending 1200 ms after it, thus equaling 3s segments in total. A baseline correction using the 200 ms pre-stimulus was applied. Finally, an automatic artifact rejection for maximum voltage steps of 50 μV/ms, amplitudes exceeding ±95 μV and activity below 0.5 μV was used.

### EEG time–frequency analysis

After averaging, time–frequency-information was extracted at the electrode Fz (this is the standard procedure used by our lab in many prior studies with this paradigm^8,15,17,26^) for the frequencies from 2 to 50 Hz using a complex Morlet wavelet (25 frequency steps on logarithmic scale, Morlet parameter c = 6, Gabor normalization). In order to assess power changes relative to the pre-stimulus baseline a normed output correction using the time-frame 200 ms prior to stimulus presentation was used. To assess theta-activity, the wavelet layer with the central frequency of 5.1 Hz (range: 4.4–5.8 Hz) was extracted and markers were set for the maximum peak power within the time-frame of 200–900 ms post-stimulus. This procedure was repeated before averaging over trials in order to reveal a single-trial oscillatory theta power time course for use in the EEG-informed fMRI analysis. With regard to high-beta-activity, the wavelet layer with the central frequency of 25.5 Hz (range: 22–29.1 Hz) was extracted and markers were set for the maximum peak power within the time-frame of 100–500 ms post-stimulus.

Statistical analysis was carried out using the software R^43^. Effects of group (controls vs. patients), valence (gain vs. loss) and magnitude of feedback (25 vs. 5 points) were assessed via a mixed ANOVA using the ez package^44^. Dependent variable was the peak theta or high-beta value and group was treated as a between-subjects factor and valence and magnitude as within-subject factors.

### fMRI acquisition

MR images were acquired with a 3-Tesla Prisma MR scanner (Siemens, Munich, Germany; software version VE11C) using a 64-channel head coil. Twenty-five slices were recorded using a standard gradient echo-planar imaging (EPI) T2*-sensitive sequence for functional blood-oxygen-level dependent (BOLD) imaging. Within each of the four blocks, there were 530 volumes (TR = 2 s; TE = 25 ms; FOV = 216 mm; matrix = 108 × 108; interleaved slice acquisition; slice thickness = 3 mm; interslice gap = 1 mm). A high-resolution (voxel size 1 × 1 × 1 mm) T1-weighted anatomical image (MPRAGE) was acquired for each subject in the same position as the EPI images. To reduce effects of spatial distortion additional fieldmaps (40 slices, TR = 634 ms; TE_1_ = 4.92 ms; TE_2_ = 7.38 ms; FOV = 224 mm; interleaved slice acquisition; slice thickness = 3 mm) were obtained.

### fMRI pre-processing

Processing of the fMRI data was conducted using the in-built functions of the Statistical Parameter Mapping software (SPM12)^45^. Fieldmaps were used to calculate voxel displacement maps (VDMs), which were used during the realignment and unwarping step. Following this, slice timing correction was applied, the images were co-registered to a common space (Montreal Neurological Institute) and spatially smoothed with an 8 × 8 × 8 mm Gaussian Kernel.

Preceding the standard pre-processing procedures as described above we visually inspected the T1-weighted anatomical images in order to exclude severe pathological alterations and checked the co-registration between pre-processed functional files and anatomical images for misalignments for each of the participants. Additionally, we assessed the amount of overall motion. To this end, we extracted the maximum translation and rotation for each block per participant and averaged this. The mean maximum translation for the control group was 0.88 mm (SD = 0.36 mm; range = 0.42—1.73 mm) and the mean maximum rotation for this group was 0.016 rad (SD = 0.007 rad; range = 0.006–0.034 rad). The mean maximum translation for the patient group was 1.25 mm (SD = 0.73 mm; range = 0.26–2.88 mm) and the mean maximum rotation was 0.019 rad (SD = 0.008 rad; range = 0.007–0.036 rad). There was no significant difference between the two groups regarding both translation and rotation. For the whole sample we identified 43 volumes that superseded a scan-to-scan translation of 1.5 mm or a rotation of 0.02 radians, thus affecting approximately 0.05% of our total volumes (maximum within one participant was 0.9%). We included the motion parameters to the GLM to control for possible movement artefacts.

### fMRI analysis

BOLD responses elicited by the feedback stimuli were modelled using the general linear model (GLM) approach. The following regressors on the subject-level were modelled before convolution with a canonical hemodynamic response function (HRF; cf. Andreou et al. 2017)^15^: 1. initial presentation of the stimulus; 2. motor response; 3. anticipation phase; 4. the four feedback conditions (maximum gain, minimum gain, maximum loss, minimum loss); 5. presentation of the account balance. All regressors were modelled as stick functions at onset of the respective phase. Additionally, 6 motion parameters were added as regressors of no interest. All subject-level contrasts for the feedback conditions were calculated as compared to the baseline.

Group-level statistics were assessed via a Full Factorial Model with three factors (group, valence and magnitude of feedback). Effects observed at a significance level of *p* < 0.05 corrected for multiple comparisons at the whole brain level (family-wise error rate (FWE)) are reported as significant. All effects are reported as *t*-contrasts (e.g. controls > patients and patients > controls for the factor group), except for the triple interaction of group x valence x magnitude. Since there were no significant effects including the factor magnitude in the EEG analysis or in the triple interaction in the regular fMRI analysis and the EEG-informed fMRI analysis this factor will not be further addressed in the following.

### fMRI region of interest (ROI) analysis

Based on prior literature regarding the processing of loss feedback both in healthy participants^15^ and BPD patients^8^ we defined the ACC (bilateral) as an anatomical region of interest (ROI) for the fMRI analysis. To this end we used the SPM Wake Forest University Pickatlas toolbox (Version 3.0.5)^46^ to create an image file of the ACC (“Anterior Cingulate” (bilateral) mask in the “TD Labels” section of the human atlas). This was used as a mask to extract the whitened and filtered data for each subject in this region from one-sample *t*-Tests for the four feedback conditions (i.e. maximum gain, minimum gain, maximum loss, minimum loss) contrasted against the baseline. These were then averaged per subject for each of the feedback conditions and the resulting data was analyzed using a mixed 2 (Group) × 2 (Valence) × 2 (Magnitude)-ANOVA design from the ez package^44^ in R^43^.

### EEG-informed fMRI analysis

The effect of coupling the theta oscillatory power with the BOLD response was investigated using a separate GLM. For each of the four feedback conditions (see fMRI analysis) a parametric modulator representing the single-trial oscillatory theta power at electrode Fz was added to the single-subject GLM. Beforehand, the single-trial oscillatory theta power had to be checked for possible artefacts. To this end, the individual subjects’ extracted data was checked for amplitudes exceeding the mean value for each condition in each block by more than 2.5 SD. These values were replaced with the individual median value for the condition in that block. This affected 4.27% of overall trials. This procedure was based on earlier studies by our group^47^.

The parametric regressors were orthogonalized with respect to the original regressors, using the built-in orthogonalization function. This was done to remove shared variance between the regressors and parametric modulators.

Group-level statistics were assessed via a Full Factorial Model with three factors (group, valence and magnitude). Because of the known low signal-to-noise ratio in EEG-fMRI coupling analyses a more tolerant threshold compared to the regular fMRI analysis was applied and effects observed at *p* < 0.005 (uncorrected) with a cluster extent of *k *≥ 100 voxels are reported as significant (cf. Andreou et al., 2017)^15^.

### Correlation analysis

Correlations between impulsiveness assessed via the BIS^25^ and regions showing significant activation for the factors group, valence or the interaction of these two factors in the aforementioned fMRI analyses were calculated using a ROI approach. To this end we created ROI-masks of the clusters that emerged as significant from the fMRI analysis or the EEG-informed fMRI analysis using the SPM MarsBar toolbox (Version 0.44)^48^. One exception from this procedure was the ROI within the ACC, where the same ROI was used as the one used for the fMRI ROI analysis (see above) because this form of ROI analysis does not delineate any peak voxels. Activation values from all ROIs were extracted with the same method described in the fMRI ROI analysis section (see above) and incorporated into the correlational analysis in R^43^. Pearson correlation coefficients are reported.

## Supplementary Information

Supplementary Information.

## Data Availability

Data will be made available via the Open Science Framework platform upon publication.
