# Supplementary material for: Simultaneous EEG-fMRI reveals theta network alterations during reward feedback processing in borderline personality disorder
Source: Sci Rep. 2021 Aug 30;11:17336. doi: 10.1038/s41598-021-96209-7 (PMC8405642; doi:10.1038/s41598-021-96209-7)
Supplement: Supplementary file 1 — Supplementary Information. [file 41598_2021_96209_MOESM1_ESM.pdf]

Simultaneous EEG-fMRI reveals theta network alterations during reward feedback processing in borderline personality disorder

Paul A. Schauer, Jonas Rauh, Sarah V. Biedermann, Moritz Haaf, Saskia Steinmann, Gregor Leicht, and Christoph Mulert

Supplementary information

Results

EEG time-frequency analysis

Table S1. EEG-Results: Theta frequency range (electrode Fz, central frequency of 5.1 Hz, time-frame 200-900ms post-stimulus)

Effect	F(1,35)	p
Group	0.895	0.351
Valence	4.074	0.051
Magnitude	0.704	0.704
Group x Valence	4.382	**0.044**
Group x Magnitude	0.803	0.376
Valence x Magnitude	0.077	0.783
Group x Valence x Magnitude	0.036	0.851

Abbreviations: EEG, electroencephalography

Table S2. EEG: Feedback-related evoked theta power (electrode Fz, central frequency of 5.1 Hz, time-frame 200-900ms post-stimulus)

	Gain	Loss
	Mean (SD)	Mean (SD)
Controls	5.52 (6.25)	11.97 (14.52)
Patients	6.64 (7.77)	6.61 (10.33)

Abbreviations: EEG, electroencephalography; SD, standard deviation

Table S3. EEG: Feedback-related evoked theta peak latency (electrode Fz, central frequency of 5.1 Hz, time-frame 200-900ms post-stimulus)

	Gain	Loss
	Mean (SD)	Mean (SD)
Controls	460 ms (208 ms)	457 ms (200 ms)
Patients	471 ms (197 ms)	508 ms (247 ms)

Abbreviations: EEG, electroencephalography; SD, standard deviation

Table S4. EEG-Results: High-beta frequency range (electrode Fz, central frequency of 25.5 Hz, time-frame 100-500ms post-stimulus)

Effect	F(1,35)	p
Group	0.366	0.550
Valence	1.225	0.276
Magnitude	0.882	0.354
Group x Valence	0.098	0.756
Group x Magnitude	2.410	0.130
Valence x Magnitude	0.874	0.356
Group x Valence x Magnitude	1.434	0.239

Abbreviations: EEG, electroencephalography

Table S5. EEG: Feedback-related evoked high-beta power (electrode Fz, central frequency of 25.5 Hz, time-frame 100-500ms post-stimulus)

	Gain<br>Mean (SD)	Loss<br>Mean (SD)
Controls	3.91 (4.71)	4.87 (4.02)
Patients	3.79 (2.80)	4.33 (2.48)

Abbreviations: EEG, electroencephalography; SD, standard deviation

fMRI region of interest (ROI) analysis

To locate the observed effect within the ACC ROI from the regular fMRI analysis we additionally conducted an exploratory analysis using the built-in ROI analysis function of SPM. We assessed the statistics via a Full Factorial Model with three factors (group, valence and magnitude of feedback) and used the created image file of the ACC (bilateral) to mask the data. We then checked the loss > gain contrast and set a significance level of FWE < 0.05 (see Figure S1 below). We found an effect in the right dACC with a cluster extend of k = 2.

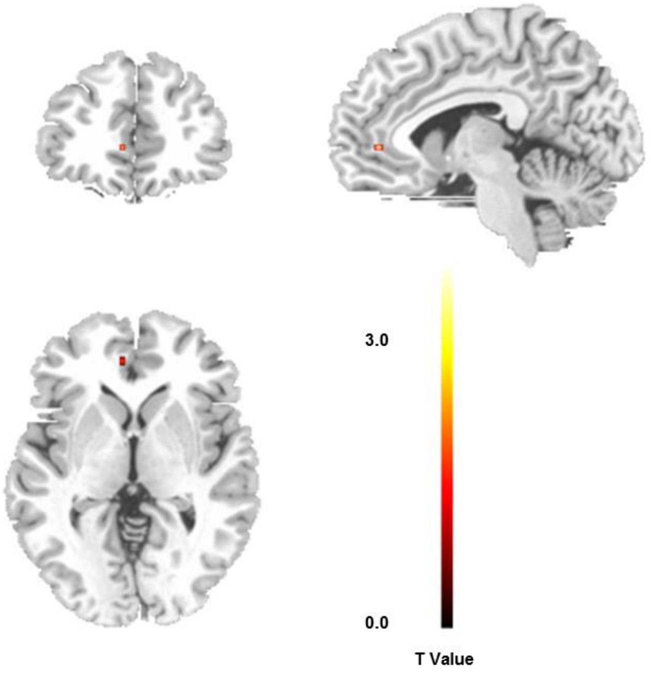

Figure S1. Areas showing stronger BOLD-responses for loss vs. gain in the ACC ROI (regular fMRI; significance level: p(FWE) < 0.05). This figure was created using SPM12[1] (Version 12; www.fil.ion.ucl.ac.uk/spm/software/spm12/) and MRIcro[2] (Version 1.40; people.cas.sc.edu/rorden/mricro/).

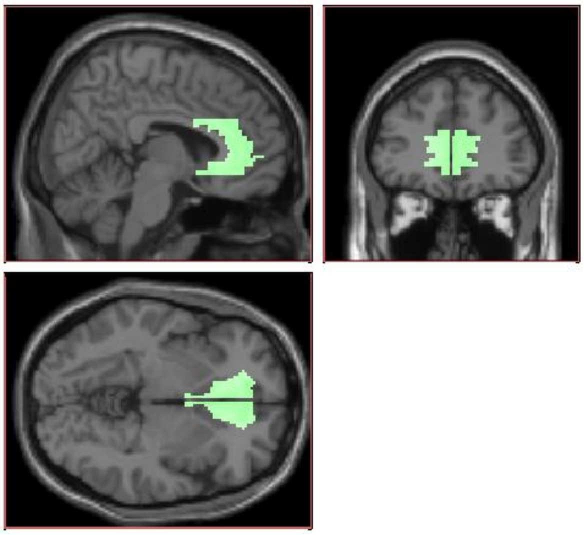

Figure S2. Mask of the ACC ROI constructed with the SPM Wake Forest University Pickatlas toolbox (Version 3.0.5)[3] and used during the fMRI region of interest (ROI) analysis. This figure was created using SPM12[1] (Version 12; www.fil.ion.ucl.ac.uk/spm/software/spm12/).

Correlation analysis: Correlation plots

The following correlation plots illustrate the correlations between the subjects' impulsivity ratings and the specific feedback related activity (i.e., the whitened and filtered data for each subject in this region from one-sample t-Tests for the four feedback conditions (i.e. maximum gain, minimum gain, maximum loss, minimum loss) contrasted against the baseline). General feedback related activity and loss related activity were calculated by averaging the extracted values across the respective conditions.

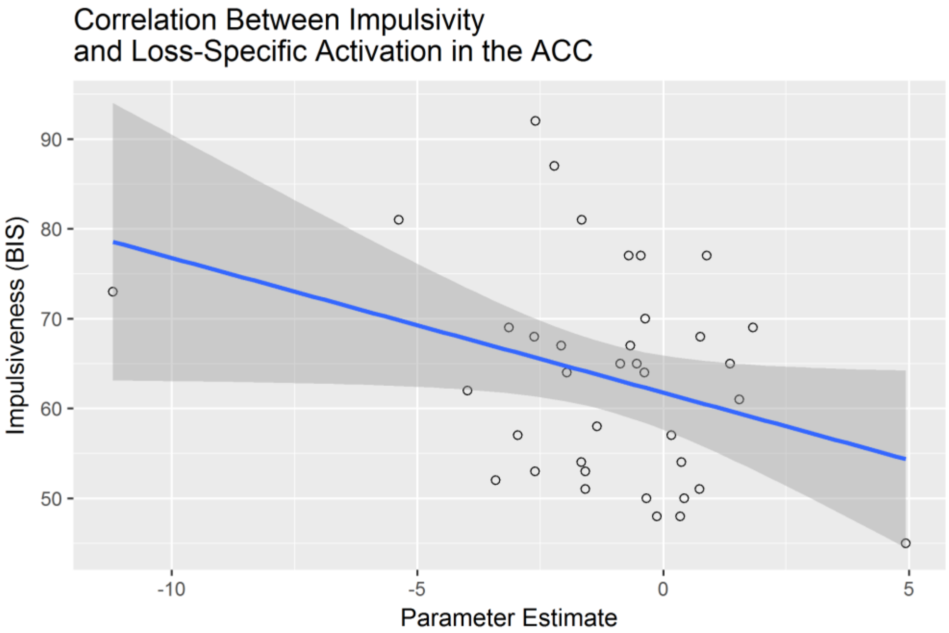

Figure S3. Plot depicting the correlation between impulsiveness (assessed via the BIS) and loss related activity in the ACC ROI (regular fMRI analysis).

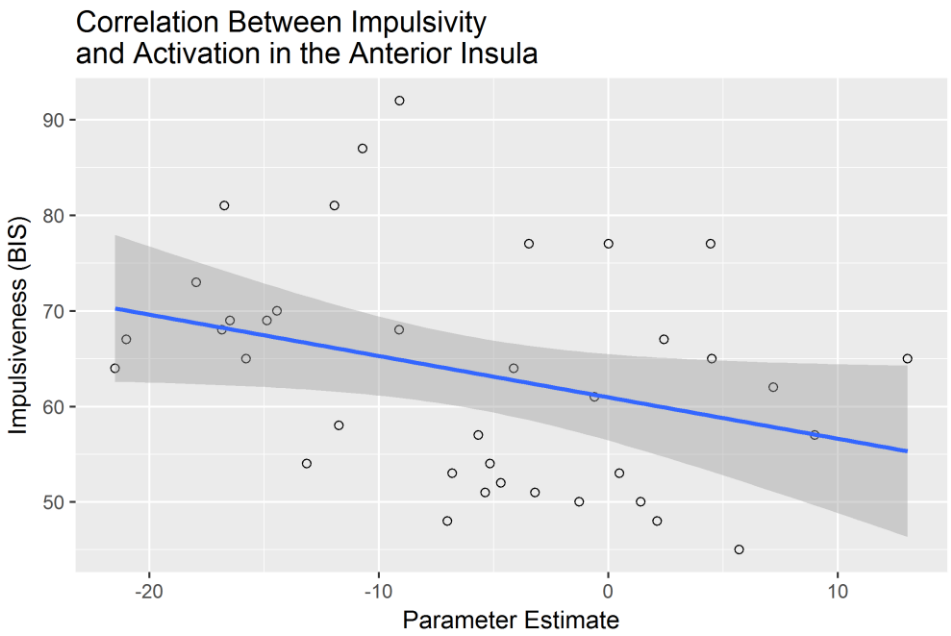

Figure S4. Plot depicting the correlation between impulsiveness (assessed via the BIS) and general feedback related activity in the anterior insula (regular fMRI analysis).

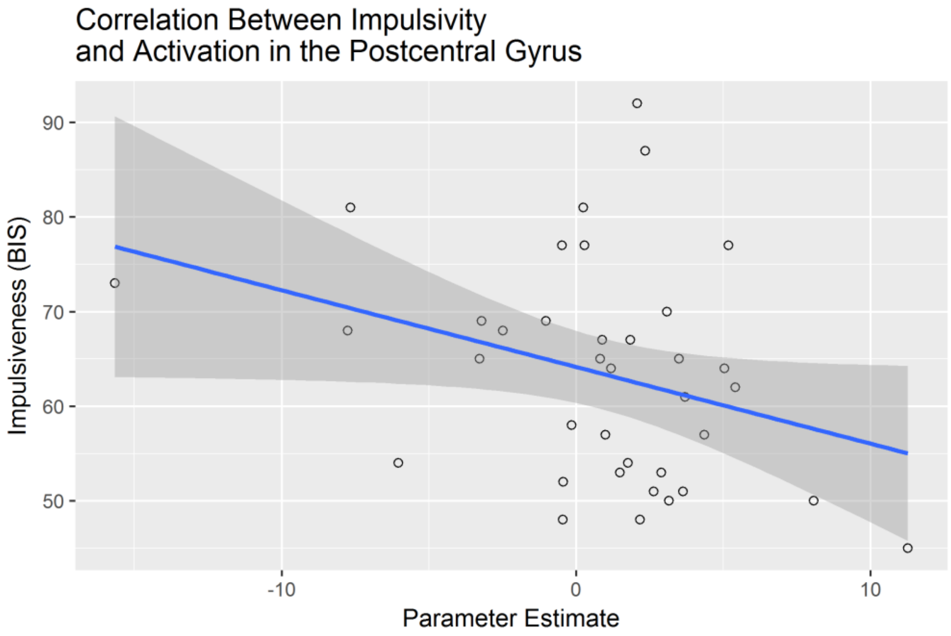

Figure S5. Plot depicting the correlation between impulsiveness (assessed via the BIS) and general feedback related activity in the postcentral gyrus (regular fMRI analysis).

Helium pump comparison

To assess the effect of the fMRI scanners helium pump on the EEG in the frequency bands we investigated, we gathered data from one participant, who was placed in the scanner with the same

set-up used for all participants of the study. We conducted one measurement whilst the helium pump was switched on and one whilst it was switched off (both 5 minutes in length). The data was then analyzed using the Brain Vision Analyzer software (Version 2.1, Brain Products). The following pre-processing steps were applied to the data: 1. Changing the sampling rate to 250 Hz; 2. Filtering (0.1 Hz low-pass; 120 Hz high-pass); 3. Automatic raw data inspection; 4. Segmentation (2 s); 5. Fast-Fourier transform; 6. Averaging. This pilot data from one participant is depicted in Figure S6.

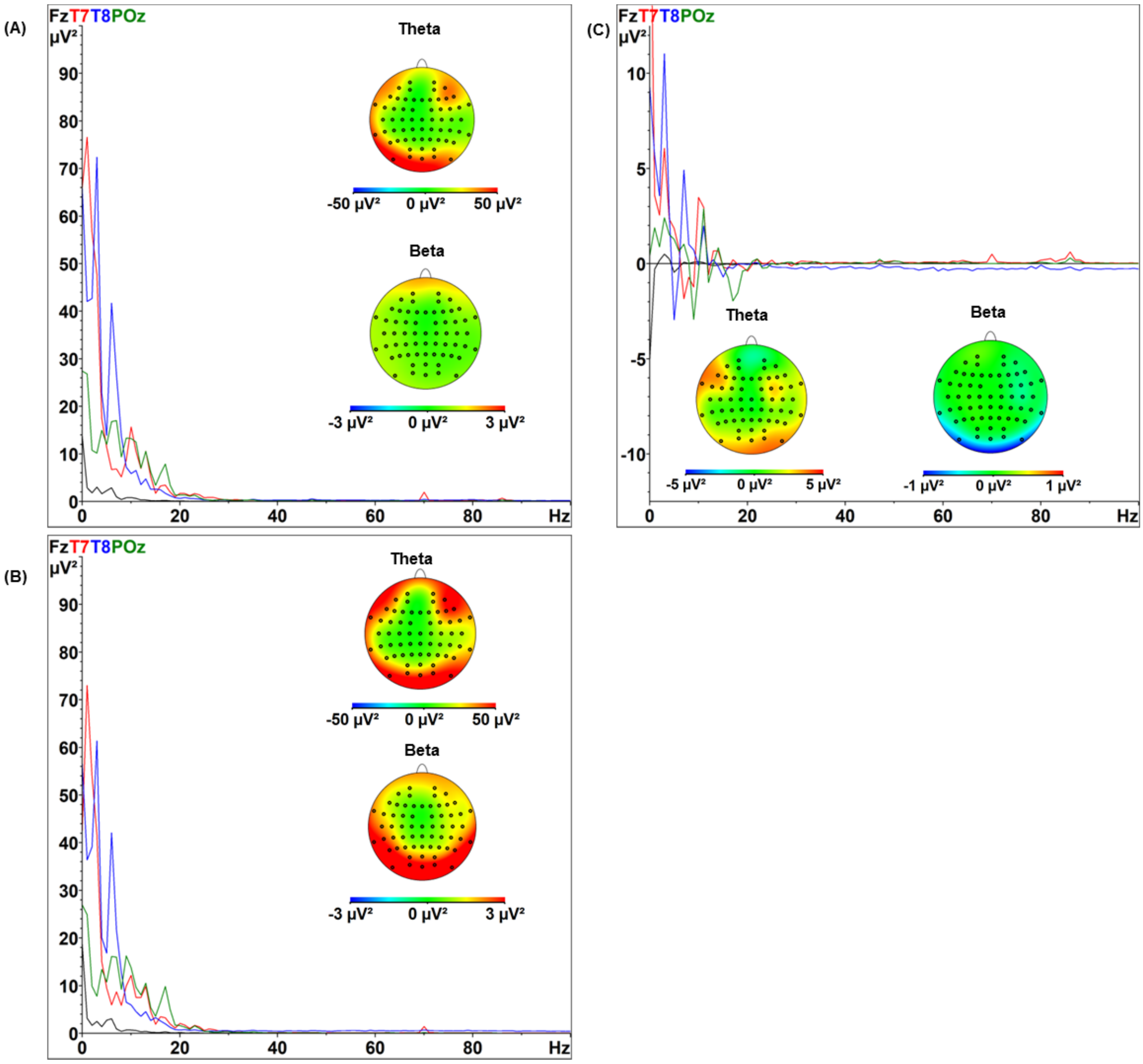

Figure S6. Plot depicting the power for the frequencies between 0.1 Hz and 100 Hz with the helium pump switched on (A), switched off (B) and the difference between the two (C) at the electrodes Fz (black), T7 (red), T8 (blue) and POz (green). Topographical maps for the theta (3.5 – 7.5 Hz) and beta (12.5 – 30 Hz) frequency bands were added to the plot.

References

1 Statistical Parameter Mapping Software 12 (London, UK, 2014).
2 Rorden, C. & Brett, M. Stereotaxic display of brain lesions. *Behavioural neurology* **12**, 191-200 (2000).
3 Maldjian, J. A., Laurienti, P. J., Kraft, R. A. & Burdette, J. H. An automated method for neuroanatomic and cytoarchitectonic atlas-based interrogation of fMRI data sets. *Neuroimage* **19**, 1233-1239 (2003).
